# CRISPRing the hypertrophic cardiomyopathy: correcting one pathogenic variant at a time

**DOI:** 10.1038/s41392-023-01526-0

**Published:** 2023-06-26

**Authors:** Junaid Afzal, Kshitiz

**Affiliations:** 1 Department of Medicine, Division of Cardiology, University of California San Francisco, San Francisco, CA 94158 USA; 2 Department of Biomedical Engineering, University of Connecticut Health, Farmington, CT 06032 USA

**Keywords:** Cardiology, Molecular medicine, Medical genetics

In recent paired Nature Medicine publications, Reichert et al.^1^ and Chai et al.,^2^ adopted the Crispr/Cas9-adenine base editing (ABE) approach to base correct one of the most common pathogenic variant of hypertrophic cardiomyopathy (HCM) — Myh7 gene (c.1208 G > A: p.Arg403Gln). They delivered^1,2^ ABE-Cas9 with single guide RNA (sgRNA) via intra-thoracic injection of dual-AAV9 virus which resulted in the dramatic reduction of the hypertrophy and fibrosis in the adult mice model of HCM.

Familial HCM is the most common cardiac genetic disease that affects 1 in 500 people.^3^ It has a characteristic feature of left ventricular hypertrophy with spatially localized myocyte hypertrophy, disarray, and fibrosis—a classical histopathological hallmark, which cannot be explained by hypertension or abnormal loading conditions.^3^ HCM is also one of the most common causes of sudden cardiac death in the young population, and presents with progressive cardiac fibrosis and heart failure, which ultimately requires heart transplantation.^3^ It arises from autosomal dominant variants of sarcomeric genes, and while over 40% of variants are still unidentified, one-third of the known pathological variants are present in the myosin heavy chain 7 (Myh7) gene, which encodes beta myosin heavy chain (βMHC) of thick filament, an essential protein in cardiomyocytes contractility. Most of the mutations are clustered around specific hotspots like myosin mesa, converter domain, and the proximal S2 region of βMHC.^4^

**Fig. 1:**
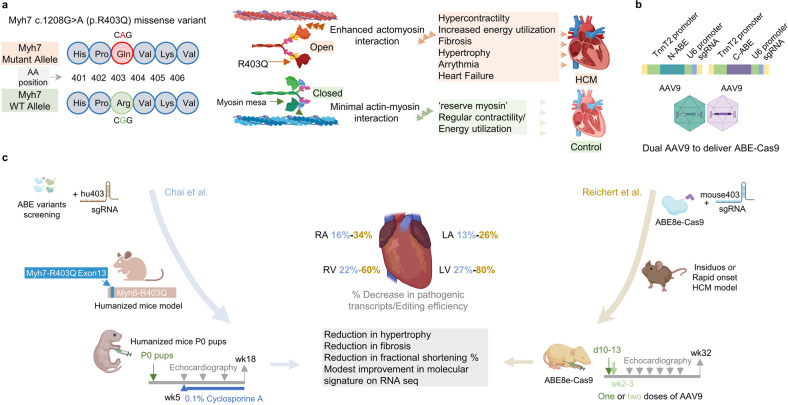
**a** The heterozygous Myh7 gene mutation causes open myosin conformation which results in increased myosin partnership with actin (N_a_) with accompanying hypercontractility and higher energetic cost. This results in the progressive worsening of the disease phenotype. **b** Both studies employed the dual AAV9 to deliver ABE-Cas9. **c** Chai et al.^2^ (left-blue arrow) screened the ABE variants with a human sgRNA to choose the variant with minimal bystander/off-target effect. They generated a humanized mice model with the incorporation of the human Myh7 region in the orthologous Myh6 mice gene. Mice pups at PO were injected with AAV9. Cyclosporine A was used to precipitate the disease phenotype. Reichert et al. (right-brown arrow) used ABE8e with sgRNA complementary to the mouse R403Q region in two different R403Q mice models. They injected the mice at d 10–13 (single) or with a second dose of AAV9 at week 2–3. Both studies showed improvement in disease phenotype with correction of pathogenic variants (blue: Chai et al, brown: Reichert et al.), enhanced contractility on echocardiography, and reduced fibrosis on histology. Modest improvement was observed on molecular signature. Created with BioRender.com

The heterozygous pathological variant of Myh7 (p.R403Q) is perhaps the most characterized variant of HCM with a dominant negative effect. The mutation causes a loss of positive charge in the mesa region of βMHC which results in a higher number of myosin heads in open configuration, thereby increasing their interaction with actin. This ultimately results in the hypercontractility of myocytes and the high energy cost of each cardiac cycle with resultant progressive phenotype (Fig. 1a). Although, recent pharmacological approaches, using cardiac myosin inhibitors during the established disease phase have shown some reduction in the disease phenotype by reducing the muscle contractility, it is also associated with reduced systolic function and may result in the worsening of heart failure. Crispr/Cas9-adenine base editing (ABE) system offers an alternate approach where the known HCM mutations can be corrected before the disease phenotype is established. In the ABE system, the adenosine deaminase, fused to a catalytically impaired Cas9, is taken to a ‘target window’ by sgRNA to achieve A-to-G base substitutions, without DNA double-strand breaks.^5^ It has been successfully utilized in several genetic diseases and offers efficient and precise correction with significantly lower off-target effects.^5^ But it is limited by the necessity of adenine base presence at a specific distance from the PAM site in the editing (target) window. Although the presence of ‘non-target adenine’ in the editing window can also produce a bystander editing effect which can generate new pathological variants, this effect is low due to the site-PAM distance specificity of ABEs.^5^

Reichert et al.^1^ used ABE8e under cardiac troponin promoter to limit the delivery of base editor to the cardiomyocytes (Fig. 1b). They used their mice model of R403Q-129SvEv and R403Q-129SvEv/S4 mice which have an insidious or a rapid onset/fulminant HCM phenotype respectively. They observed a significant reduction in the disease phenotype with a single injection of ABE-AAV9 at 10–13 days of age, in both mice (Fig. 1c). While the editing efficiency was significantly lower in atria (~30%) compared to ventricles (~70%), indicating a chamber-specific editing effect, they did observe a modest increase in editing efficiency in atria with a second dose of AAV injection with an increase in bystander editing. They also compared the efficiency of HCM mutant allele silencing, using AAV9 delivery of Cas9 nuclease under TnnT2 promoter, and observed deleterious consequences which indicate the advantage of the ABE system over traditional gene silencing approach using Cas9.

In comparison, Chai et al.^2^ adopted a more humanized mouse model approach (Fig. 1c). They first screened variants of ABEmax (narrow window) and ABE8e (wide window but highly processive) in human induced pluripotent stem cells (iPSCs), and found high bystander editing with ABE8e. Using ABEmax-VRQR with a targeting sgRNA in iPSCs, they found >98% editing efficiency and rescue of the disease phenotype in mutant iPSCs-derived cardiomyocytes. As mice model of R403Q are orthologous HCM models with a mutation in the Myh6 gene, Chai et al.^2^ generated a more relevant humanized mice model using a portion of the human Myh7 gene around the pathogenic variant (Fig. 1c). This model offers the additional benefit of testing the on-target efficacy and the bystander editing effect of the ABE-sgRNA system, using a human pathogenic region, in a preclinical setting. Using a single intrathoracic injection of AAV9 containing ABEmax under the cardiac specific TnnT promoter and a human sgRNA, they reported ~30% reduction in pathological variants with improvement in contractile function and reduction in cardiac fibrosis and pathological hypertrophy (Fig. 1b, c). Although they observed similar AAV9 transduction efficiency in different chambers of the heart, they also reported the reduced atrial editing efficiency like Reichert et al.^1^

Previously, Ma et al.^6^ reported an in-utero delivery of AAV9-ABEmax to base correct the R403Q mutation and observed an improvement in the disease phenotype. These studies^1,2,6^ offer incremental evidence supporting the utilization of the ABE system for known HCM mutations. It is pertinent to note that all the reported studies^1,2,6^ have shown the rescue of disease phenotype by editing 30–60% of pathological variants of R403Q. The dominant negative nature of the R403Q variant warrants the comprehensive functional and molecular assessment of myocytes to evaluate the effect of variable editing on the disease phenotype. The RNA seq data does indicate a modest improvement in molecular data and reversal of some ontologies. Similarly, both studies show the chamber-specific reduction of pathogenic variants, with a significantly lower editing efficiency in atria. HCM is known to present with atrial fibrosis and fibrillation. It is important to dissect the role of gene editing in rescuing the phenotype within each chamber, with an additional focus on parameters like energetics and electrophysiology along with contractility and fibrosis. The presence of high pathological variants in the atrial chamber could adversely affect the pathophysiology of the disease with unintended consequences. While ventricular hypertrophy and fibrosis can be improved by the partial removal of pathological variants from the ventricles. Still, the persistent atrial disease can cause progressive atrial fibrosis and fatal arrhythmia if left untreated. Additionally, a significant challenge to AAV therapy is the presence of natural immunity in 30–60% of human population, which is dramatically increased with a single injection of AAV vectors. This immune challenge makes it difficult to clinically translate the AAV therapies in the current form.

Aside from the limitations mentioned above that need to be addressed in future studies, the overall improvement of cardiac function with minimal off-target/bystander effects indicated by both studies suggests the potential utility of the ABE-Cas9 system in cardiac genetic diseases like HCM.
